# The parent drugs chloroquine and hydroxychloroquine do not inhibit human CYP3A activity in vitro

**DOI:** 10.1007/s00228-020-02928-7

**Published:** 2020-06-18

**Authors:** Xia Li, Rainer Höhl, Fritz Sörgel, Uwe Fuhr

**Affiliations:** 1 Faculty of Medicine and University Hospital Cologne, Center for Pharmacology, Department I of Pharmacology, University of Cologne, Gleueler Straße 24, 50931 Cologne, Germany; 2 Institute for Clinical Hygiene, Medical Microbiology and Clinical Infectiology, Paracelsus Medical Private University, Nuremberg Hospital, Nuremberg, Germany; 3 IMBP-Institute for Biomedical and Pharmaceutical Research, Nürnberg, Heroldsberg Germany; 4 Institute of Pharmacology, West German Heart and Vascular Centre, University of Duisburg-Essen, Essen, Germany

To the Editor,

Among 796 clinical trials to treat COVID-19, chloroquine and hydroxychloroquine account for a large fraction with 46 trials for chloroquine and 120 trials for hydroxychloroquine (https://clinicaltrials.gov/ as of 24 April 2020). Despite the lack of reliable clinical data, due to their significant inhibitory effects on viral cell entry and replication [1], both drugs have been recommended to treat patients diagnosed as mild, moderate, and severe cases of COVID-19 pneumonia [2]. However, for critically ill patients, co-medications are usually required. Unfortunately, there is little information on potential drug-drug interactions caused by chloroquine and hydroxychloroquine.

During compassionate treatment of two adult COVID-19 patients with hydroxychloroquine (day 1: 2 × 400 mg, thereafter 2 × 200 mg daily) and clarithromycin (2 × 500 mg daily), routine therapeutic drug monitoring on days 2 and/or 3 of treatment showed unexpectedly high clarithromycin concentrations (> 10 mg/L around the end of infusion). The patients were on mechanical ventilation but had no renal failure and were of normal body weight. Clarithromycin was given for suspected bacterial superinfection to cover atypical pathogens of a possible community-acquired pneumonia. The finding indicates that hydroxychloroquine may inhibit cytochrome P450 (CYP)3A, since clarithromycin is primarily metabolized by CYP3A [3]. In addition, hydroxychloroquine was reported to inhibit CYP2D6-mediated metabolism of metoprolol in vivo [4], and chloroquine also decreased CYP2D6 activity [5]. However, there is no data on a potential inhibition of CYP3A4 by chloroquine and/or hydroxychloroquine.

Therefore, an in vitro assay to assess inhibition of CYP3A4 by the two drugs was performed using a published method (see supplementary materials of reference [6]). The formation of 1′-hydroxymidazolam from midazolam was used as the CYP3A4 probe reaction, as recommended by the FDA and EMA [7, 8]. A 250 mg chloroquine phosphate tablet (Avloclor® 250 mg tablets, Alliance Pharma PLC, Wiltshire, UK) or a 200 mg hydroxychloroquine sulphate tablet (Quensyl® 200 mg tablets, Sanofi-Aventis, Paris, France) was dissolved by the addition of 2 L of water to obtain the respective stock solutions. *K*_*i*_ values were determined to assess the effect of chloroquine and hydroxychloroquine on CYP3A4 by incubating a range of substrate (i.e., 0.2, 0.6, 2,6, 12, 20 μM) and inhibitor concentrations (0, 0.6, 1.25, 2.5, 5, 10, 20, 40 μM) with 1.85 pmol/ml CYP3A4 for 8 minutes. The assays were carried out in duplicate. The resulting metabolite was quantified by LC-MS/MS as described [6]. Datasets were analyzed using GraphPad Prism 7 (GraphPad, La Jolla, CA, USA) [9].

**Fig. 1:**
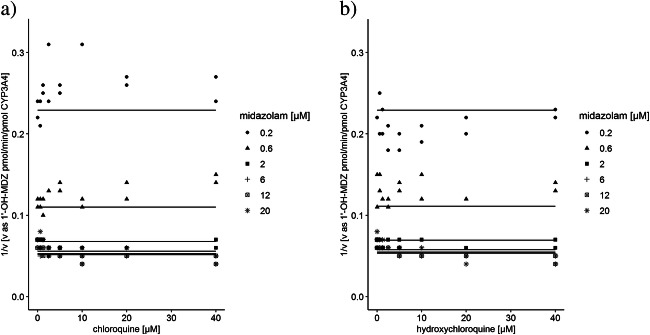
Dixon plot of in vitro assay for inhibition of CYP3A4 by chloroquine (**a**) and hydroxychloroquine (**b**). v, enzyme activity rate. 1′-OH-MDZ, 1′-OH-midazolam. Lines indicate the fits according to the competitive inhibition model obtained from the entire dataset

*K*_m_ and *V*_max_ values for midazolam hydroxylation in the two inhibition assays were very similar, i.e., 0.72 μM and 20.0 pmol 1′-OH-MDZ/min/pmol CYP3A4 for the chloroquine experiment, and 0.69 μM and 19.4 pmol 1′-OH-MDZ/min/pmol CYP3A4 for hydroxychloroquine. For midazolam concentrations below 2 μM, there was no apparent effect of a range of concentrations of chloroquine and hydroxychloroquine on CYP3A4 activity (Fig. 1). At higher midazolam concentrations, enzyme activity showed a trend to increase with higher concentrations of both chloroquine and hydroxychloroquine. While the mechanism for this observation is unknown, clearly there was no inhibitory effect. The goodness of fit indicated that the nonlinear competitive inhibition model described the data reasonably well, and also respective *K*_*i*_ values (i.e., 9.18 × 10^95^ μM for chloroquine and 1.14 × 10^88^ μM for hydroxychloroquine) approaching infinity clearly showed that both drugs did not cause inhibitory action on CYP3A4.

The current in vitro assay demonstrated that chloroquine and hydroxychloroquine do not inhibit CYP3A4 activity, excluding the possibility that the parent drugs cause the observed high clarithromycin exposure by this mechanism. However, we cannot exclude whether metabolites of these drugs may inhibit CYP3A. The reason for high clarithromycin concentrations when co-administered with hydroxychloroquine should be further explored.
